# Solidification-Induced Formation of 3D Ge Frameworks in Al–Ge Alloy Microparticles

**DOI:** 10.3390/ma19102153

**Published:** 2026-05-21

**Authors:** Olha Khshanovska, Vladyslav Ovsynskyi, Aleksandr Kryshtal

**Affiliations:** Faculty of Metals Engineering and Industrial Computer Science, AGH University of Krakow, 30-059 Krakow, Poland; khshanov@agh.edu.pl (O.K.); ovsynskyi@agh.edu.pl (V.O.)

**Keywords:** Al–Ge microparticles, rapid solidification, undercooling, eutectic network, sponge-like Ge, phase separation, irregular eutectics

## Abstract

**Highlights:**

- Al–Ge microparticles were deeply undercooled and solidified on Si, SiO_2_, Al_2_O_3_, a-C, and ZrO_2_ substrates.
- Morphology, structure and composition of separated phases were analyzed using SEM, TEM, EDX, and EELS.
- The wetting angle of Al–Ge microparticles varied from 46° to 123°, depending on the substrate.
- Solidification on all substrates studied produced a single coral-like Ge network embedded in an Al matrix.

**Abstract:**

The solidification behavior and microstructural evolution of Al–Ge microparticles were investigated on Si, SiO_2_, Al_2_O_3_, amorphous carbon, and ZrO_2_ substrates. Micrometer-sized particles with a hypereutectic composition were produced by melting and resolidifying 40 nm thick Al–Ge films. Their size, wetting angle, crystal structure, and internal morphology were characterized by SEM and TEM techniques. We demonstrate that Al–Ge particles exhibited strongly substrate-dependent wetting, with contact angles ranging from 46° on SiO_2_ to 123° on ZrO_2_. Nevertheless, all particles developed a similar internal microstructure consisting of a fully interconnected, irregular Ge network within an Al matrix, indicating complete phase separation during solidification. The eutectic network was quantified by its ligament thickness. No correlation was found between ligament thickness and substrate type or contact angle, indicating that the coral-like internal Ge network forms independently of particle wetting. Instead, the ligament thickness increased with particle size and during post-solidification annealing. The network gradually coarsened up to 310 °C, followed by its complete breakdown and transformation into an equilibrium Janus morphology at 370 °C. These findings provide new insight into the solidification of irregular eutectic systems and suggest a route for tailoring three-dimensional internal microstructures in eutectic microparticles.

## 1. Introduction

Complex three-dimensional structures, such as sponge- or coral-like architectures at the nano- and microscale, have attracted significant attention due to their potential for practical applications. For example, inorganic nanoporous materials are used in drug delivery systems and diagnostics [1,2]. In addition, their catalytic activity has found applications in CO_2_ reduction [3,4,5]. They are also employed in energy conversion and storage: dye-sensitized solar cells, electrochromic windows, and batteries [6]. Within this class of materials, germanium-based 3D structures have emerged as particularly promising anode materials for next-generation lithium-ion batteries [7,8].

In practice, porous three-dimensional architectures can be obtained either by assembling particles [5,9] or by selectively removing one component from an alloy to create a porous network [10,11]. For example, the Al–M (M=Au, Pd, Pt, and Ag) alloys were prepared by induction melting and subsequently fabricated into thin ribbons by melt spinning in an argon atmosphere. These ribbons were then subjected to chemical dealloying in hydrochloric acid (HCl), during which Al was selectively dissolved, resulting in nanoporous noble metal structures [10]. Also, Adams et al. [12] demonstrate that porous morphologies can be obtained in Al–Ge thin films, where co-deposition on Si substrates leads to phase separation into Al- and Ge-rich regions at temperatures 150–375 °C. Subsequent chemical etching removes the Al-rich phase, exposing a porous Ge network. More recently, approaches in which porous structures are created within individual particles have also gained attention. For example, nanoporous gold nanoparticles were produced via solid-state dewetting of Au-Ag bilayers followed by chemical etching of Ag in Nitric acid (HNO_3_) [13].

Notably, many of the above-mentioned alloy systems employed for the fabrication of porous structures are irregular eutectic systems, where one phase is faceted and the other is non-faceted. Solidification of irregular eutectics can develop a wide range of microstructures and often lead to complex and non-periodic morphologies. Also, high solidification rates significantly influence microstructure evolution in irregular eutectics. They can be achieved by techniques such as laser melting, melt spinning, or undercooling of the melt [14]. Nevertheless, our understanding of the solidification pathways and resulting microstructure in irregular eutectics remains limited. Furthermore, a model capable of describing the formation and diversity of microstructures in irregular eutectics under highly nonequilibrium conditions is still lacking [15].

In contrast to bulk materials, where large undercooling is difficult to achieve, nano- and micron-sized particles can experience substantial undercooling [16]. As a result, highly undercooled particles enable very high solidification rates, allowing access to non-equilibrium regimes and the formation of diverse eutectic morphologies. For example, studies of Al–Ge particles in free-fall conditions in a drop tube revealed that for undercooled particles with sizes ranging from 100 µm to 1000 µm, a structural evolution from a eutectic laminar to a eutectic anomalous structure occurred [17].

Accordingly, this work investigates the morphology, phase structure, and internal eutectic network of solidified Al–Ge microparticles formed on various substrates.

## 2. Materials and Methods

**Figure 1:**
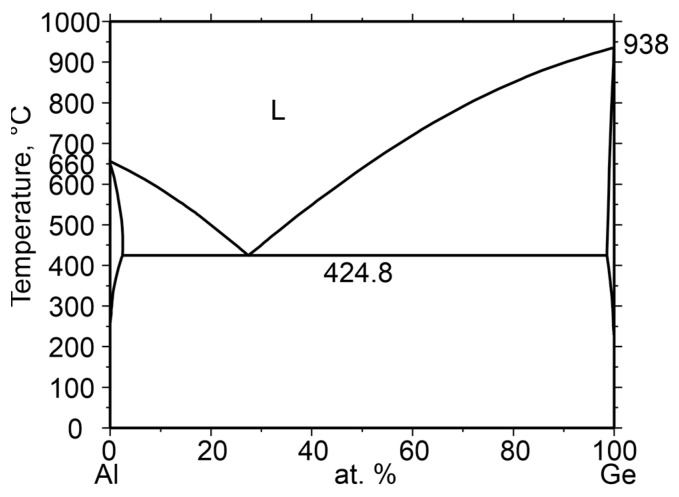
Equilibrium phase diagram of the Al–Ge system.

The Al–Ge system exhibits a simple binary eutectic phase diagram (Figure 1) with negligible mutual solid solubility of Al and Ge. The eutectic reaction occurs at 424.8 °C at 28 at.% Ge. This system belongs to the class of irregular eutectics. According to the Jackson roughness factor, defined as [18]:

$$\alpha =\frac{∆H_{m}}{RT_{m}},$$

where $∆H_{m}$ is the enthalpy of melting, R is the gas constant, and $T_{m}$ is the melting temperature. Materials with (α<2) exhibit non-faceted growth, whereas materials with (α>2) tend to form faceted interfaces. The Jackson factors are $\alpha_{Al}\approx 1.38$ and $\alpha_{Ge}\approx 3.16$, indicating non-faceted growth for Al and faceted growth for Ge.

A series of Al–Ge film samples was prepared on various 5 × 5 mm^2^ substrates, including Si, SiO_2_, Al_2_O_3_, C, and ZrO_2_. Commercial Si chips (Ted Pella, Inc., Redding, CA, USA; product no. 16008) were used as the base substrate. Identical Si chips with a thermally grown 300 nm SiO_2_ layer were also employed. For the carbon-coated variant, an approximately 15 nm thick amorphous carbon layer was deposited onto the Si chips to serve as a diffusion barrier. Single-crystal sapphire (Al_2_O_3_, c-plane (0001), thickness 0.5 mm; SPI Supplies, West Chester, PA, USA), double-side polished and used as received, was included as another substrate. Finally, tetragonal yttria-stabilized zirconia (3Y-TZ) substrates were fabricated by pressing commercially available powder (TZ-3Y-E, Tosoh, Tokyo, Japan) and subsequently polished. The ZrO_2_ substrates were machine-polished using Diamond Pads and MD-Polishing Cloth (Struers, Cleveland, OH, USA) with 1 μm diamond paste to achieve a mirror-like surface [19].

The Al–Ge film depositions were carried out at room temperature using a DC magnetron sputtering system (PRIMS 032, PREVAC, Rogów, Poland). Each film was deposited in two consecutive steps using high-purity (99.999%) Al and Ge targets (Kurt J. Lesker Company, Jefferson Hills, PA, USA). A 26 nm Al layer was followed by a 14 nm Ge layer, resulting in a total nominal thickness of 40 nm and an overall composition of approximately 29 at.% Ge. The base pressure in the deposition chamber was 2 × 10^−6^ mbar. The argon gas pressure was 2.5 × 10^−3^ mbar. After deposition, the samples were annealed at 550 °C for 20 min under vacuum, which led to the formation of Al–Ge microparticles through thermal dewetting and eutectic melting of Al–Ge films.

The morphology and internal structure of the crystallized Al–Ge microparticles were examined using scanning electron microscopy (SEM). Imaging was carried out on a Zeiss Merlin field-emission SEM (Carl Zeiss Microscopy, Oberkochen, Germany) operated at an accelerating voltage of 7.5 kV. Secondary electron (SE) and angle-selective backscattered electron (AsB) detectors were used to visualize surface morphology and compositional contrast, respectively. Ligament thickness was determined from top-view SEM images using Fiji software (ImageJ, version 1.54p) [20] with the BoneJ [21] plugin. After image binarization and thresholding to separate the phases, the ligament thickness distribution was estimated using the thickness function, and the mean value with standard deviation was calculated for several representative particles on each substrate.

A cross-sectional lamella was prepared from a selected microparticle using focused ion beam (FIB) milling. The procedure was carried out using ZEISS Crossbeam 350 Gemini dual-beam system (Carl Zeiss Microscopy, Oberkochen, Germany). The lamella cut was transferred to a copper half-grid and analyzed using a probe Cs-corrected Titan Cubed G2 60-300 (Thermo Fisher Scientific, Waltham, MA, USA) electron microscope operated at 300 kV. Imaging was conducted in scanning transmission electron microscopy (STEM) mode using a high-angle annular dark-field (HAADF) detector (Model 3000, E.A. Fischione Instruments, Inc., Export, PA, USA ). EDX map, covering an area of 13 × 12 µm^2^ (1007 × 923 pixels, ~12 nm/pixel), was acquired over 6400 s using a beam current of 200 pA. Low loss and core-loss EELS spectra were recorded using a Gatan Quantum GIF 963 spectrometer (Gatan, Inc., Pleasanton, CA, USA) with a beam current of 300 pA, a convergence semi-angle of 13 mrad, and an energy dispersion of 1 eV/channel. The spectra were processed in Gatan DigitalMicrograph by background subtraction and removal of multiple scattering to isolate the single-scattering core-loss signal, and the relative elemental composition was determined using the software’s built-in quantification tools with Hartree–Slater cross-sections. Structural characterization was supported by selected area electron diffraction (SAED), and the diffraction patterns were indexed with the help of the Java Electron Microscopy Software (JEMS, version 5.1931u2024b22) [22].

## 3. Results

Melting of a 40 nm thick Al–Ge film resulted in the formation of Al–Ge particles with sizes ranging from submicrometer to several micrometers, regardless of the substrate used. However, the composition, shape, and wetting angle of the particles varied over a wide range depending on the substrate type and particle size. In this work, we focused on single particles shaped as spherical segments with sizes larger than 3 µm, which form through melting and subsequent solidification of hypereutectic Al–Ge alloy.

**Figure 2:**
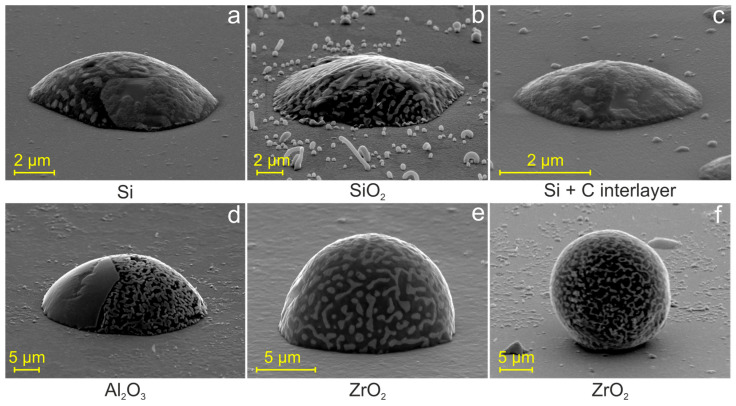
Secondary electron SEM images of Al–Ge particles formed on different substrates, acquired at a 70° tilt: (**a**) Si, (**b**) SiO_2_, (**c**) Si with a carbon interlayer, (**d**) Al_2_O_3_, and (**e**,**f**) ZrO_2_.

Figure 2 shows representative Al–Ge particles on various substrates imaged at a 70° tilt. Contact angles were estimated from particle geometry using the relation [23]:

$$H_{k}=R\left[1+sin(\theta -\alpha )\right]=\frac{d}{2sin\theta }\left[1+sin(\theta -\alpha )\right],$$

where $H_{k}$ is the apparent particle height, radius of curvature R, d (for θ<90°) is the particle diameter, and α= 70° corresponds to the SEM stage tilt angle used during imaging. Assuming the primary sources of uncertainty are particle asymmetry, ambiguity in edge definition, and local deviations from spherical-segment geometry, the estimated accuracy of the contact angle is approximately ±5°.

On Si substrates, the contact angles ranged from 52° to 75°, whereas on SiO_2_ they were consistently lower, approximately 46°. Depositing a thin carbon film on Si had little effect, yielding contact angles of 46–56°. On Al_2_O_3_ (sapphire) substrates, the particles exhibited a contact angle of ≈79°. However, the situation changed dramatically on the ZrO_2_ substrate, where contact angles exceeded 90°, ranging from 96° to 145°. This indicates a non-wetting behavior, reflecting the inert nature of ZrO_2_ substrate and the lack of significant interaction with Al–Ge melt.

Additionally, Figure 2 reveals a complex multiphase microstructure in the Al–Ge microparticles. All particles exhibit an irregular, interconnected Ge network within the Al matrix. Some particles (Figure 2a,d) also contain solid Ge-rich regions, indicating that they were not fully liquid at 550 °C and that their solidification proceeded from a two-phase (solid + liquid) region of the phase diagram. Notably, the microstructure pattern of individual particles was fully reproducible upon resolidification, indicating that the solidification pathway is highly deterministic.

**Figure 3:**
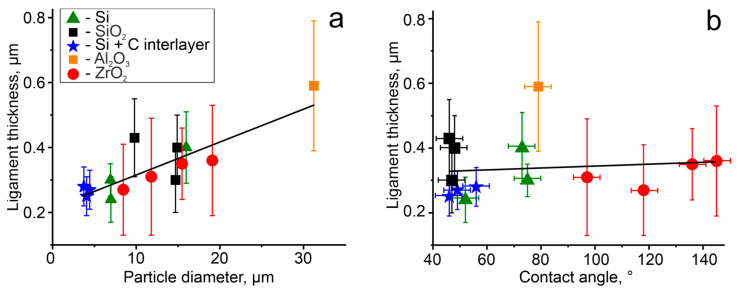
Dependence of ligament size on particle diameter (**a**) and contact angle (**b**). The legend in graph (**a**), indicating the substrate type, applies to both graphs. Solid black lines represent linear fits to the experimental data.

We characterized the eutectic network in several particles on different substrates using ligament thickness (Figure 3). No apparent correlation was observed between the mean ligament thickness and the particle–substrate contact angle over the range of 46° to 145° (Figure 3b). However, ligament thickness tends to increase with particle size, with larger particles exhibiting thicker ligaments (Figure 3a).

**Figure 4:**
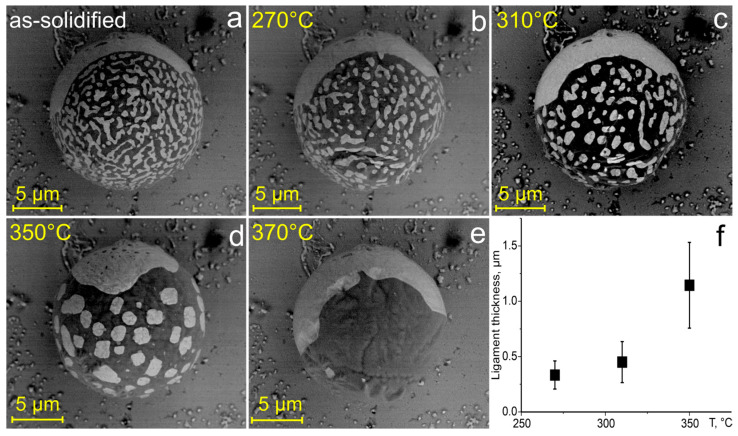
(**a**–**e**) BSE-SEM images of the same Al–Ge particle on ZrO_2_ substrate: (**a**) as-solidified; (**b**–**e**) after 20 min annealing at the temperatures indicated in the upper left corner. (**f**) Dependence of ligament thickness on temperature.

To study the thermal evolution of the eutectic network, a selected Al–Ge particle on ZrO_2_ substrate was annealed at different temperatures for 20 min. Before each annealing, the particle was resolidified to restore the initial eutectic network. Figure 4a–e shows SEM images of the same Al–Ge particle after annealing, while Figure 4f shows the corresponding dependence of ligament thickness on annealing temperature. These data reveal gradual coarsening of the network up to 310 °C (Figure 4a–c). At higher temperatures, the irregular network breaks down completely (Figure 4d), and at 370 °C the particle adopts an equilibrium Janus morphology with distinct Al- and Ge-rich regions (Figure 4e).

The temperature dependence of ligament coarsening in the Al–Ge particles over the range 270–350 °C was analyzed using an Arrhenius plot of the natural logarithm of ligament thickness versus inverse temperature (1/T). A linear fit to the data yields an activation energy of 117 ± 22 kJ/mol.

**Figure 5:**
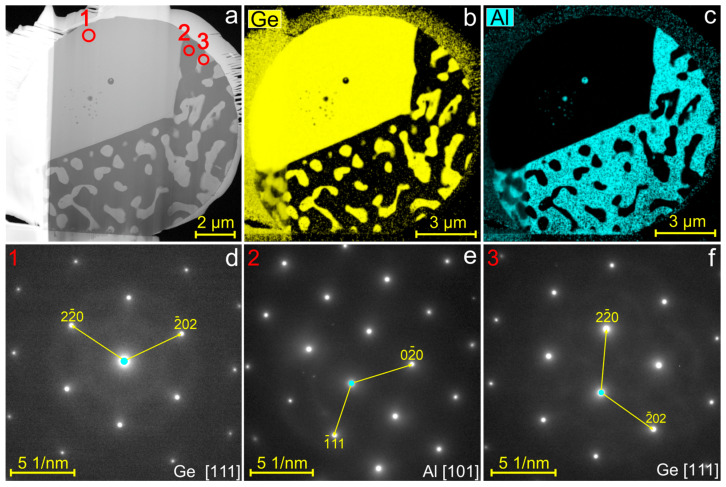
(**a**) HAADF-STEM image and (**b**,**c**) EDX elemental maps of the Al–Ge particle lamella. (**d**–**f**) SAED patterns obtained from regions 1–3 in (**a**), respectively. The patterns were indexed to (**d**) Ge [111], (**e**) Al [101], and (**f**) Ge [111] lattices.

The crystalline structure and composition of the phases constituting the Al–Ge particles were investigated in TEM using thin lamellae cut from the middle of a particle. Figure 5 shows a HAADF-STEM image of lamella with three representative regions analyzed by SAED. The diffraction patterns exhibit sharp reflections corresponding to single-crystalline Ge in the [111] zone axis and single-crystalline Al in the [101] zone axis. The calculated lattice parameters, 4.06 ± 0.025 Å for Al and 5.68 ± 0.025 Å for Ge, match well with reference values (Al: 4.05 Å [24]; Ge: 5.65 Å [25]).

**Figure 6:**
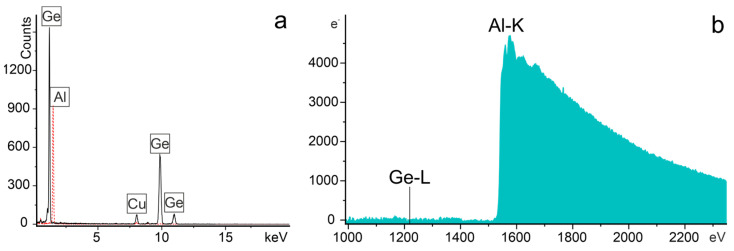
(**a**) EDX spectra acquired from regions 1 (black solid line) and 2 (red dashed line) in Figure 5 of the Al–Ge particle lamella; (**b**) EELS spectrum acquired from the Al-rich region of the particle (region 2 in Figure 5).

The EDX elemental maps (Figure 5b,c) clearly reveal compositional separation into Ge-rich and Al-rich regions. EDX quantification shows that the Al-rich regions contain up to 5 at.% Ge, whereas the Ge-rich regions contain up to 3 at.% Al. The representative spectra acquired from the Ge-rich and Al-rich regions of the particle are shown in Figure 6a. At the same time, the weak Ge signal observed in EDX may be influenced by X-Ray signal delocalization due to electron scattering effects, therefore we acquired core-loss EELS spectrum from Al region of the lamellae (Figure 6b). The spectrum exhibits a pronounced Al-K edge at ~1560 eV, while no Ge-L_2,3_ edge (~1217 eV) is detected. Thus, diffraction and elemental analyses show that the Al–Ge particles consist of nearly pure single-crystalline fcc Al and diamond-cubic Ge domains, indicating that complete phase separation occurred during solidification.

**Figure 7:**
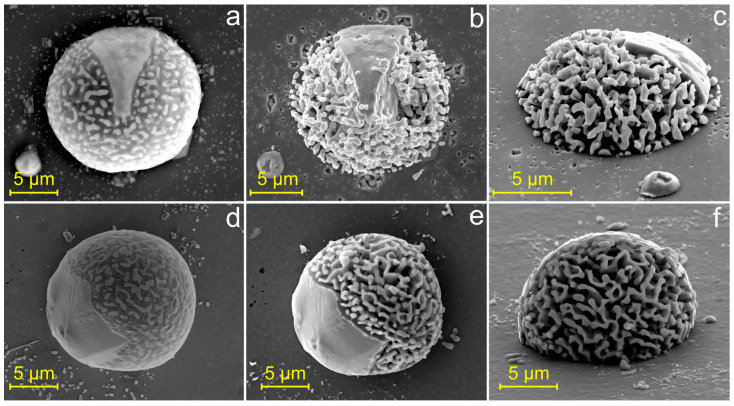
SEM images of Al–Ge microparticles on Si (**a**–**c**) and ZrO_2_ (**d**–**f**) substrates. Images (**a**,**d**) show the particles before etching, whereas images (**b**,**c**) and (**e**,**f**) show the same particles after chemical etching. Images (**c**) and (**f**) were acquired at tilt angles of 53° and 70°, respectively.

Since Al is readily dissolved in hydrochloric acid while Ge remains intact, chemical etching was employed as a simple and effective method to visualize the internal eutectic network of the particles. Figure 7a shows a representative particle on a Si substrate before etching, whereas Figure 7b,c present the same particle after selective etching. A similar approach was applied to particles formed on a ZrO_2_ substrate (Figure 7d–f). An Al–Ge particle on ZrO_2_ substrate with relatively low contact angle was selected to ensure mechanical stability of the three-dimensional microstructure after Al etching.

Selective etching revealed a single, fully interconnected Ge network formed during solidification of the Al–Ge microparticles. Notably, the same type of Ge network was observed on both Si and ZrO_2_ substrates, despite their substantially different wetting angles.

## 4. Discussion

Our study demonstrates the formation of an irregular, fully interconnected Ge network in solidified Al–Ge microparticles. Surprisingly, the characteristic size of the eutectic network in solidified hypereutectic Al–Ge microparticles does not depend on the contact angle or substrate type (Figure 3b). Specifically, a ligament size of 0.2–0.5 μm was observed in particles with contact angles ranging from 46° to 145° (Figure 2 and Figure 3). The formation of a “coral-like” network morphology is indicative of rapid eutectic solidification. In our experiments, significant melt undercooling was required to achieve the high growth velocities necessary for the formation of interconnected networks. Therefore, all microparticles investigated in this work solidified at nearly the same temperature.

Classical nucleation theory predicts that the solidification temperature of an undercooled liquid particle on a substrate is strongly influenced by the contact angle, as it governs the efficiency of heterogeneous nucleation. For a particle on a substrate, the nucleation energy barrier is reduced by a factor *f*(*θ*) relative to homogeneous nucleation:

$$∆G_{het}^{*}=f\left(\theta \right)∆G_{hom}^{*},$$

where

$$f\left(\theta \right)=\frac{1}{4}\left(2+cos\theta \right){\left(1-cos\theta \right)}^{2}$$

Experimentally, the effect of the contact angle on the magnitude of undercooling during solidification of metal nanoparticles on various substrates has been investigated, for example, in Ref. [26]. It has been shown that the degree of undercooling scales with the contact angle of the particle–substrate system, reaching approximately one-third of the bulk melting temperature at $\theta \approx 150^{\circ }$, which corresponds to the limit of homogeneous nucleation. In the case of alloy nanoparticles, the liquidus temperature is used to quantify the undercooling during solidification. Therefore, our observations cannot be explained within the framework of classical nucleation theory [27], highlighting the need for alternative approaches to describe the solidification pathways of Al–Ge microparticles.

In this context, a nonclassical solidification pathway previously reported for the sister irregular eutectic Au–Ge system [28,29] may provide a suitable framework for interpreting the present results. It was shown that solidification of highly undercooled single-phase nanoparticles proceeds in a two-stage manner over a temperature range of up to 70 °C. This behavior is attributed to a chemical depletion effect [30] and to constraints on nucleation and growth of structurally distinct phases within the limited volume of the nanoparticles. The onset of solidification in alloy particles occurs at temperatures characteristic of heterogeneous nucleation, whereas the terminal solidification temperature is significantly lower, approaching values associated with homogeneous nucleation. As a result, much larger melt undercoolings can be achieved in nano- and microparticles of irregular eutectic alloys prior to complete solidification. Furthermore, it was demonstrated that two-phase (solid + liquid) nanoparticles can be undercooled to a similar extent as single-phase liquid particles prior to solidification.

These results are consistent with the observations of the present study. Solidified hypereutectic Al–Ge particles exhibit the same type of eutectic network, irrespective of substrate type. SEM images of these particles frequently reveal large Ge crystals, suggesting that some of these particles were not fully melted at 550 °C but instead contained a solid Ge phase, i.e., existed in a two-phase state. Furthermore, the relatively small contact angles observed on Si, SiO_2_, Si+C, and Al_2_O_3_ substrates indicate a possible interaction between the liquid Al–Ge alloy and the substrate. As a result, partial dissolution of Si, Al, or oxygen into the melt may occur, introducing impurities that reduce the nucleation barrier. Nevertheless, all particles solidified under conditions of significant undercooling, as inferred from their morphology.

Importantly, the solidified Al–Ge particles exhibit a single, fully interconnected network, which provides a strong indication of constraints on the solidification pathway and, in particular, on the nucleation kinetics. First, an interconnected Ge-rich skeleton spanning the entire particle volume is most consistently explained by single-site nucleation followed by continuous growth, rather than multiple independent nucleation events. Indeed, if multiple Ge nuclei were activated within the droplet, one would expect the formation of several competing growth domains separated by impingement boundaries, which were not observed.

From a kinetic standpoint, this suggests that the first successful nucleation event rapidly depletes the available undercooled liquid before additional nuclei can form. Owing to its higher melting temperature, and keeping in mind that Ge is a faceted phase, it leads the solidification and nucleates first [14], most likely heterogeneously at the particle–substrate interface where the nucleation barrier is minimized. As solidification proceeds, Ge phase presumably grows into the undercooled liquid under diffusion-limited conditions, while nucleation of the Al phase appears constrained due to its higher energy barrier. Consequently, the remaining liquid would become highly undercooled and strongly enriched in Al. Once a critical state is reached, this residual liquid undergoes rapid, nearly simultaneous solidification. The solidification second stage may not involve independent nucleation events. Instead, the pre-existing Ge phase could act as a structural and crystallization seed, guiding the solidification of the Al-rich liquid, which spreads up the faceted phase. As a result, the transformation would propagate through the entire remaining volume in a correlated manner, producing a single, continuous, and fully interconnected network.

We were not able to directly measure the solidification temperature range in the particles; however, the second-stage solidification temperature could be assessed from the data of Figure 4f, which shows the dependence of the ligament size on the annealing temperature. Indeed, ligament evolution during annealing is governed by coarsening, which is a pure diffusional process, while the solidification sets the initial scale. Since the measurable increase in the ligament size was observed in the particle annealed at 270 °C, the solidification temperature must be lower than this value. Assuming that post-solidification coarsening is primarily governed by volume diffusion, and applying the measured activation energy of 117 ± 22 kJ/mol, the Arrhenius law gives an estimated solidification temperature for the Al–Ge particles of 243 ± 6 °C. This temperature is 181 °C lower than the eutectic temperature of 424 °C in the Al–Ge system. Consequently, the relative supercooling of liquid Al–Ge microparticles reaches ≈0.26T_e_, which is significantly higher than the reported value of ≈0.13T_l_ (where T_l_ is the liquidus temperature) in hypoeutectic Al_70_Ge_30_ alloy subjected to rapid solidification [31].

The slight dependence of the characteristic size of the eutectic network on the size of solidified Al–Ge microparticles should be discussed. It is well known that the cooling rate depends on particle size. For a spherical particle of radius *R*, the characteristic cooling time *t* can be approximated as [32]:

(1) t=R2α,

where *α* is the thermal diffusivity.

For eutectic solidification, the Jackson–Hunt relation [33] gives the following scaling law:

$$\lambda^{2}V=const,$$

where *λ* is ligament size and *V* is the growth velocity. Though it is valid for the classical steady eutectic growth, for undercooled particles the eutectic network size still scales with particle size [34]. Hence, higher cooling rates, and consequently faster growth, produce finer eutectic networks in the particles. As a result, particle size provides an additional means of tuning the eutectic network scale alongside supercooling effects. This observation is consistent with studies on microstructure size in undercooled Al–Ge droplets [17].

## 5. Conclusions

This work studies the microstructures formed during the solidification of undercooled Al–Ge irregular eutectic alloy microparticles on Si, SiO_2_, Al_2_O_3_, C, and ZrO_2_ substrates. We demonstrate that regardless of the substrate wetting conditions (contact angle below or above 90°), all particles exhibit a similar internal microstructure, forming a fully interconnected and irregular Ge network within an Al matrix. No apparent correlation was observed between the ligament thickness and the particle–substrate contact angle. However, ligament thickness increased with particle size, with larger particles generally exhibiting thicker ligaments. TEM analysis, combining SAED, EDX, and EELS, revealed complete phase separation during solidification, resulting in nearly pure, single-crystalline fcc Al and diamond-cubic Ge domains. By analyzing the temperature dependence of ligament coarsening, the solidification temperature of the microparticles was estimated to be approximately 243 °C, which is significantly lower than values reported for rapidly solidified alloys. The solidification pathway was not consistent with classical nucleation theory, suggesting constraints on the nucleation and growth of structurally distinct phases within confined Al–Ge particle volumes.

## Data Availability

The original contributions presented in this study are included in the article. Further inquiries can be directed to the corresponding author.
